# Surgical management of petrosal dural arteriovenous fistulas

**DOI:** 10.3171/2025.7.FOCVID2598

**Published:** 2025-10-01

**Authors:** Ari D. Kappel, Sahin Hanalioglu, Anil Can, David I. Bass, Rose Du

**Affiliations:** Department of Neurosurgery, Mass General Brigham, Brigham and Women’s Hospital, Harvard Medical School, Boston, Massachusetts

**Keywords:** dural arteriovenous fistula, cerebrovascular, skull base, vascular malformation, petrosal, retrosigmoid

## Abstract

Dural arteriovenous fistulas (DAVFs) are rare with a low incidence. Tentorial DAVFs account for 10%–15% of all intracranial DAVFs and are associated with an aggressive clinical course. Petrosal DAVFs are a subtype of tentorial DAVFs and account for approximately 26% of tentorial DAVFs. They are supplied by the marginal tentorial artery or external carotid artery branches and drain into the petrosal vein and/or the superior petrosal sinus. Petrosal DAVFs may be challenging to treat endovascularly, while surgical management is often relatively safe and effective. Here the authors present two cases of surgically managed petrosal DAVFs.

The video can be found here: https://stream.cadmore.media/r10.3171/2025.7.FOCVID2598

## Transcript

### 0:24 Background.

Dural arteriovenous fistulas or DAVFs are rare, with a low incidence in the population of approximately 0.16 cases per 100,000 person years.^1^ Tentorial DAVFs account for approximately 10%–15% of all intracranial dural AV fistulas.^2–4^ They are associated with an aggressive course and are dangerous if ruptured.^2–4^ Petrosal dural AV fistulas, or Lawton type 5 tentorial dural AVFs, drain into the petrosal vein and/or superior petrosal sinus and account for approximately 26% of tentorial dural AVFs.^2^

The marginal tentorial artery of Bernasconi and Cassinari is the most common arterial supply to petrosal dural AVFs.^2,5^ The middle meningeal artery is also a common source of arterial supply and other ECA branches including the ascending pharyngeal artery and occipital artery may be seen.^2,5^ They drain into the petrosal vein or superior petrosal sinus. Here, you can see a left common carotid artery injection of a petrosal dural AVF supplied by the marginal tentorial artery from the meningohypophyseal trunk of the left ICA draining to the petrosal vein.

### 1:23 Treatment Options.

Petrosal DAVFs may be challenging to treat endovascularly because of the small size of arteries supplying them and the risk of off-target embolization of important structures, including the internal carotid artery and its circulation and cranial nerves including the facial nerve.^6^

Surgical management is often relatively safe and effective with easy access to the petrosal ridge and the fistulous point of the dural AV fistula from a retrosigmoid approach.^2,6^ Here, we present two cases of surgically managed petrosal DAVFs.

### 1:50 Case 1 – Presentation.

A 56-year-old female with past medical history of multiple sclerosis, hypertension, hyperlipidemia, presented with sudden onset, severe left-sided headache, nausea, and vomiting. CT of the head demonstrated subarachnoid hemorrhage in the posterior fossa.

On examination, the patient complained of a severe headache and was noted to have a slight left facial droop with no other significant findings. CT angiography of the head was obtained and demonstrated dilated vessels near the left tentorium.

Diagnostic cerebral angiography demonstrated a left petrosal dural AV fistula. Arterial supply from the left marginal tentorial artery of Bernasconi and Cassinari, a branch of the MHT, was seen. AP and lateral views of a left common carotid artery injection demonstrate the left petrosal dural AV fistula supplied by the left marginal tentorial artery. Venous drainage is directly to the petrosal vein with retrograde flow in the cerebellar veins. These findings are consistent with a ruptured Borden grade III left petrosal dural AV fistula.

### 2:46 Case 1 – Surgical Video.

A retrosigmoid craniotomy was performed in standard fashion. The petrosal vein was identified at the junction of the petrous ridge in the tentorial margin. Bipolar electrocautery was used to coagulate the petrosal vein. The coagulated vein was sharply cut with microscissors. Bipolar electrocautery coagulates the petrosal vein, ensuring complete obliteration of the dural AV fistula. Cranial nerve V is seen underneath the cut petrosal vein. Intraoperative video fluoroscopy with indocyanine green injection confirms complete obliteration of the dural AV fistula.

Postoperative DSA demonstrated complete obliteration of the dural AV fistula. The patient’s facial droop resolved, and she was discharged home on postoperative day 3.

### 3:45 Case 2 – Presentation.

A 33-year-old male with no significant past medical history presented with sudden onset headache and left facial droop. CTA demonstrated a left petrosal dural AV fistula supplied by the marginal tentorial artery, as well as a 1-cm partially thrombosed venous varix causing mass effect on the left cerebellar hemisphere.

Preoperative DSA demonstrated high-grade petrosal dural AV fistula with predominant feeders from the left meningohypophyseal trunk and marginal tentorial artery, as well as left external carotid artery feeders, including the middle meningeal artery, left occipital artery, and ascending pharyngeal artery. There was venous drainage to the left superior petrosal sinus and evidence of multiple posterior fossa venous varices with local mass effect and compression.

### 4:28 Case 2 – Patient Positioning and Setup.

A retrosigmoid craniotomy was planned. The patient was positioned supine with the head turned and fixed in Mayfield pins. A standard C-shaped incision was fashioned behind the left ear. Intraoperative neuromonitoring throughout the case included motor evoked potentials, somatosensory evoked potentials, brainstem auditory evoked responses, and cranial nerves V and VII.

### 4:44 Case 2 – Surgical Video.

The transverse and sigmoid sinuses were exposed. The dural edges were reflected, and CSF was drained to allow dynamic retraction of the cerebellum, providing access to the petrous ridge and tentorial margin. The petrosal vein was identified at the junction of the petrous ridge and the tentorium. Bipolar electrocautery was used to coagulate the petrosal vein and its tributaries. Microscissors were used to sharply cut the petrosal vein. Video fluoroscopy with ICG confirmed complete obliteration of the dural AVF. A watertight primary dural closure was performed and the incision was closed in standard fashion. Postoperative DSA showed obliteration of retrograde cortical venous drainage.

### 6:15 Conclusions.

Petrosal dural AV fistulas are associated with an aggressive and dangerous course. They are primarily supplied by the marginal tentorial artery and drain to the petrosal vein and superior petrosal sinus. They may be challenging to treat endovascularly because of the small arteries supplying them and the risk of off-target embolization to the internal carotid circulation or other important structures, including the facial nerve.

Surgical management of petrosal dural AV fistulas is often relatively safe and effective. They can be treated with a retrosigmoid craniotomy and division of the petrosal vein. This obliterates a Borden type III DAVF and converts a Borden type II to a Borden I dural AV fistula, which can be managed conservatively.

## Disclosures

The authors report no conflict of interest concerning the materials or methods used in this study or the findings specified in this publication.

## Author Contributions

Primary surgeon: Du. Assistant surgeon: Kappel, Bass. Editing and drafting the video and abstract: all authors. Critically revising the work: Du, Kappel, Hanalioglu, Can. Reviewed submitted version of the work: all authors. Approved the final version of the work on behalf of all authors: Du. Supervision: Du, Bass.

## Supplemental Information

### Patient Informed Consent

The necessary patient informed consent was obtained in this study.
